# Correlation between the gut microbiota composition and cognitive frailty: a case–control study in community-dwelling older adults

**DOI:** 10.3389/fnut.2025.1709481

**Published:** 2026-01-12

**Authors:** Nana Wen, Yu Zhang, Liwei Sun, Jianquan He, Guohua Zheng

**Affiliations:** 1 College of Nursing and Health Management, Shanghai University of Medicine and Health Sciences, Shanghai, China; 2 Department of Rehabilitation, School of Medicine, Zhongshan Hospital of Xiamen University, Xiamen University, Xiamen, China

**Keywords:** cognitive frailty, gut microbiota, community-dwelling older adults, case–control study, 16S rDNA

## Abstract

**Background:**

Although alterations in the gut microbiota have been documented in older adults with mild cognitive impairment or physical frailty, the relationship between gut microbiota changes and cognitive frailty (CF) which is a clinical syndrome characterized by the coexistence of physical frailty and mild cognitive impairment in the absence of any dementia, remains unclear. This study aimed to investigate the gut microbial composition associated with CF and to assess the differences between community-dwelling older adults with CF and those without CF using 16S rDNA gene sequencing.

**Methods:**

A case–control study was conducted in community-dwelling older adults. Thirty-five older adults with CF and an equal number of older adults without CF were recruited. Fecal samples were examined by 16S rDNA sequencing. Differences in microbial composition at various taxonomic levels between the two groups were identified and evaluated for correlations with cognitive or physical function.

**Results:**

The *β*-diversity differed significantly between the CF and non-CF groups. Several taxa were more abundant in the CF older adults, including the phylum *Verrucomicrobia* and *Tenericutes*, the class *Verrucomicrobiae* and *Mollicutes*, and three orders (e.g., *Verrucomicrobiales*), six families (e.g., *Akkermansiaceae*), and eight genera (e.g., *Escherichia-shigella*). In contrast, the orders *Betaproteobacteriales* and *Pasteurellales*, the families *Burkholderiaceae* and *Pasteurellaceae*, and the genera *Roseburia* and *Haemophilus* were more abundant in the non-CF older adults (all *p* < 0.05). Altered gut microbiota effectively discriminated CF older adults well from controls (AUC = 92%). Certain microbiota enriched in the CF older adults, such as *Family_XIII* and *Lactobacillaceae*, showed negative correlation with physical performance, while *Lactobacillaceae* was also negatively correlated with cognitive performance (*p* < 0.05). conversely, microbiota more abundant in the non-CF older adults, including the genera *Roseburia and Burkholderiaceae*, were positively correlated with physical performance, and genera *Roseburia and Pasteurellaceae* were positively correlated with cognitive performance (*p* < 0.05).

**Conclusion:**

This study provides insight into the role of altered gut microbiota in the pathogenesis of CF among community-dwelling older adults. Further longitudinal studies with larger sample sizes are needed to establish causal relationships.

## 1 Introduction

Cognitive frailty (CF) is defined as a heterogeneous clinical syndrome characterized by the co-occurrence of physical frailty and cognitive impairment in the absence of Alzheimer’s disease or other types of dementia (1). Recently, CF has emerged as a pivotal concept in aging research. As global populations age, it is increasingly evident that age-related decline seldom occurs in one domain. Instead, it involves complex and interconnected deteriorations in physical, cognitive, and psychosocial domains. CF captures this multidimensional intersection, offering a holistic framework for understanding the challenges and opportunities in promoting healthy aging (2). Epidemiological evidence suggests that the prevalence of CF in older populations ranges from 1 to 22%. Specifically, it is 1–4.4% among community-dwelling older adults and 10.7–22% among clinical elderly populations (3). A recent review reported a pooled prevalence of 9% in community-dwelling older adults (4). CF has been reported to be associated with an increased risk of adverse health outcomes, such as falls, disability, hospitalization, poor quality of life, and high mortality rates (5). Therefore, understanding the underlying mechanisms of CF are of considerable public health and clinical importance.

Current evidence shows that the development of CF may be influenced by several risk factors, mainly involving aging, an unhealthy lifestyle (e.g., smoking, physical inactivity), malnutrition, depression, and multimorbidity (1, 3). The underlying pathogenesis of CF involves mechanisms such as chronic inflammation, mitochondrial dysfunction, oxidative stress, neuroendocrine dysfunction and hormonal dysregulation, which collectively contribute to both cognitive decline and physical frailty (6). Given this multifactorial pathophysiology, recent research has begun to explore the potential role of the gut microbiota in mediating these processes. The microbiota-gut-brain axis (MGB), a bidirectional communication system linking intestinal microbiota with brain function, has emerged as a critical pathway linking gut health to cognitive and physical function in older adults (7). It is proposed that gut dysbiosis may disrupt brain immune homeostasis, exacerbate oxidative stress, and trigger chronic inflammation, thereby driving the progression of both cognitive decline and physical frailty (8, 9). For example, gut dysbiosis can affect brain immune homeostasis through the MGB axis and cause cerebral accumulation of amylod-*β* peptides in the brain. Disturbances in the MGB axis may contribute to cognitive impairment and increase the risk of Alzheimer’s disease (10). Moreover, the gut microbiota also plays a critical role in maintaining host physical health by regulating intestinal immune and endocrine functions, energy homeostasis, and nutrient absorption (11). A meta-analysis also reported that the composition of the gut microbiota was significantly different between frail and non-frail older adults (12). For example, one study reported increased abundance of *Collinsella* and *Butyricimonas* and decreased abundance of *α*-diversity in prefrail versus frail individuals (13); another study reported a negative association between physical frailty and gut microbiota diversity (14). Despite evidence linking gut microbiota with either cognitive decline or physical frailty, few studies have examined their combined role in cognitive frailty among community-dwelling older adults. To address this gap, we conducted a case–control study in Shanghai, China, aiming to identify gut microbial differences associated with CF.

## 2 Methods

### 2.1 Study design and participants

This case–control study recruited 70 community-dwelling older adults aged 60 years and older. The case group included 35 individuals with cognitive frailty (CF), and the control group included 35 individuals without CF. Sample size estimation was determined using G*power 3.1 software on the basis of an expressed odds ratio (OR = 2.5) of exposed dysbiosis between older adults with CF and normal older adults.

### 2.2 Inclusion and exclusion criteria

Diagnostic criteria for CF: According to the 2013 consensus of the International Institute of Nutrition and Aging and the International Geriatrics Association (15), CF should meet the following criteria: total Edmonton Frailty Scale (EFS) score ≥5; total Montreal Cognitive Assessment (MoCA) score ≤26; and Clinical Dementia Rating (CDR) score = 0.5.

Inclusion criteria were as follows: community-dwelling older adults aged 60 years and older; meeting the diagnostic criteria for CF for the case group (MoCA scores<26, CDR scores = 0.5, and EFS scores ≥5); or being older adults without cognitive impairment (MoCA scores>26 and CDR scores = 0) or physical frailty (EFS scores <5) for the control group; and providing informed consent. Exclusion criteria were as follows: suffering from a serious mental illness or an infectious disease such as ulcerative colitis or Crohn’s disease; suffering from a gastrointestinal disease with a definite diagnosis, such as diarrhea, bowel obstruction, and colon cancer; having a history of abdominal or bowel surgery; and having taken any medication that affects the structure of the intestinal flora, such as antibiotics or probiotics, in the last 3 months.

Older adults were recruited from March 2023 to July 2023 in community settings in Pudong’s new district of Shanghai city. The physical frailty, cognitive ability and dementia differentiation of older adults were assessed by the rehabilitation physicians using the EFS, MoCA and CDS scales. The demographic characteristics and covariates of participants were collected by the postgraduate students in rehabilitation using self-designed questionnaire. This study was approved by the Ethics Committee of Shanghai University of Medicine and Health Sciences (2022--WNN-012) and was conducted in accordance with the Declaration of Helsinki. Informed consent was obtained from all older adults.

### 2.3 Survey of demographic characteristics and covariates

Demographic characteristics, primarily including age, sex, BMI, marital status, income, and education, were collected using a standardized demographic data collection survey. Physical activity level was assessed using the International Physical Activity Questionnaire (IPAQ), and sleep quality and depression were assessed using the Pittsburgh Sleep Quality Index (PSQI) and the 15-item Geriatric Depression Scale (GDS-15) (16).

Nutritional status was assessed using the Mini Nutritional Assessment Short Form (MNA-SF) (17) and food frequency questionnaire (FFQ). The MNA-SF is the most widely used tool for screening and assessing nutritional risk and consists of 18 items divided into four parts: physical measurements, dietary assessment, subjective health assessment, and comprehensive assessment. The total score of the MNA-SF is 30, with scores of 24–30 indicating normal nutritional status and scores <24 indicating nutritional risk/malnutrition. Older adults’ food frequency for last 3 months was investigated using a self-design food frequency questionnaire. The questionnaire consists of frequency and intake of 13 of the most commonly consumed food groups by local residents, in which the investigated food included the rice and noodles and their products, grain crops other than rice and wheat, fish and shrimps, poultry meat and eggs, vegetables and fruits, milk and its products, meat of domestic animals, fried food and nuts, and the frequency was designed as daily, 1 ~ 2 times per week, and 3 ~ 5 times per week. Nutrients were calculated based on following formula: $\text{Nutrient intakes}=\sum_{n=1}^{i}(f_{1i}\times f_{2i}\times N_{i})$ (f1 = weight of frequency; f2 = weight of intakes, N = amount of nutrients in food).

### 2.4 Assessment of cognitive frailty

Cognitive function was assessed using the revised Beijing version of the Montreal Cognitive Assessment (MoCA) (18), which consists of 8 cognitive domains, namely, visuospatial/executive function, naming, memory, attention, language, abstraction, delayed recall, and orientation, with scores ranging from 0 to 30, and scores below 26 indicating cognitive impairment. For older adults with less than 12 years of schooling, an additional score was added to correct for educational differences. Dementia was assessed using the Clinical Dementia Rating (CDR) scale, which consists of six domains-memory, orientation, judgment and problem solving, community affairs, home and hobbies, and personal care-on a 5-point scale (19). A CDR of 0.5 indicates mild cognitive impairment but not dementia (20). Physical frailty was assessed using the modified Edmonton Frailty Scale (EFS), which assesses 9 dimensions, including cognitive ability, general health status, physical independence, social support, medication use, nutritional status, emotional perception, control, and functional performance, totaling 17 points. Higher scores indicate greater frailty, with a score ≥5 indicating physical frailty (21).

CF was diagnosed if individuals had MoCA scores of less than 26, EFS ≥ 5, and CDR = 0.5.

### 2.5 Fecal sample collection, DNA extraction

All older adults were instructed in how to collect fecal samples. Fresh fecal samples (≥500 mg) were collected on the second day after the interview using a collector with the reliable stabilizer EffcGut (22). The samples were then stored at room temperature and mailed to Changge Biotechnology (Xiamen, China). Bacterial DNA was extracted from each fecal sample, using the QIAamp Fast DNA Stool Mini Kit (Qiagen, CA, United States). DNA was eluted and stored at −20 °C before being used as a template for next-generation sequencing libraries.

### 2.6 16S rRNA gene sequencing

The V4 region of the 16S rRNA gene was amplified using the forward primer 515F (5’-GTGCCAGCMGCCGCGGTAA-3′) and the reverse primer 806R (5’-GGACTACNVGGGTWTCTAAT-3′). A mixture containing 10 μL of KAPA HiFi HotStart ReadyMix (KAPA Biosystems, United States), 0.2 μm forward and reverse primers, and 10 ng of template DNA in a total volume of 20 μL was used. PCRs were performed and PCR products were extracted using electrophoresis on 2% agarose gels and purified using the GeneJET Gel Extraction Kit (Thermo Scientific). The PCR protocol included an initial denaturation (95 °C, 3 min) followed by 30 cycles of denaturation at 95 °C for 20 s, annealing at 60 °C for 30 s, extension at 72 °C for 30 s and extension at 72 °C for 7 min.

### 2.7 Gut microbiota analysis

Sequencing libraries were generated using the TruSeq^®^ DNA PCR-Free Sample Preparation Kit (Illumina) and index codes were added according to the manufacturer’s recommendations. Library quality was assessed using a Qubit@ 2.0 fluorometer (Thermo Scientific) and an Agilent Bioanalyzer 2,100 system. Finally, the library was sequenced on an Illumina MiniSeq platform to generate 150 bp paired-end reads.

Alpha diversity analysis was conducted using the R package ‘vegan’ to calculate a suite of standard indices to assess the microbial diversity between the two samples. The observed number of OTUs (Operational Taxonomic Units) and the Chao1 estimator were used to assess total species richness. The Shannon and Simpson indices were employed to evaluate community evenness and diversity. Good’s coverage was also calculated to verify the sufficiency of the sequencing depth, confirming that the microbial communities were adequately sampled for a reliable analysis.

Sample *β* diversity was measured using the Bray-Curtis distance on the basis of an evenly rarefied OTU abundance table (sparsity depth was 127,347). Statistical differences in the measured β diversity metrics between the two groups were determined using PERMANOVA with 999 permutations, and dispersion was checked via Adonis in the R vegan package. Both univariate and multivariate PERMANOVA were performed after correcting for potential confounders.

LEfSe analysis was performed to identify taxa with differential abundance in the different groups. The reference database for taxonomic identification was SILVA, with a 97% similarity.

### 2.8 Statistical analysis

All outcomes are expressed as the means with standard deviations (SDs) for continuous variables or as numbers with percentages for categorical variables. Demographic characteristics and covariates of the case and control groups were compared using the t-test for continuous variables with the normal distribution and a Mann–Whitney test for those without. The chi-square test was used for categorical variables. Differences in *α* and *β* diversity between the two groups were compared using the non-parametric Kruskal-Wallis rank sum test for non-normally distributed data or two-sample t-test for the normally distributed data, and the comparisons were corrected using the Benjamini-Hochberg FDR procedure, with an FDR threshold of 0.05. ROC curves were used to assess effective biomarkers for CF. Correlations between the gut microbiota and physical function or cognitive function were analyzed using partial correlation mothed. All the statistical analyses were conducted using SPSS version 21 and R software (version 4.3), and a two-tailed *p* < 0.05 was considered statistically significant.

## 3 Results

### 3.1 Basic characteristics of the participants

A total of 70 community-dwelling older adults were included in the current study, with 35 older adults with CF in the case group and 35 older adults without CF in the control group. The demographic characteristics and covariates of older adults in the case and control groups are summarized in Table 1. Cognitive and physical function were significantly lower in the case group than in the control group, and the older adults in the case group had lower MoCA scores and higher EFS scores. There were no significant differences in sex, BMI, income, current smoking, current drinking, medications, comorbidities, depression, or protein, fat, or carbohydrate intake between the two groups, but older adults in the case group were older (*p* < 0.001), more likely to be single (*p* = 0.003), less educated (*p* < 0.01), less physically active (*p* = 0.01), had poorer sleep quality (*p* = 0.031), lower nutritional status (*p* < 0.001) and lower dietary fiber intake (*p* = 0.002) than those in the control group.

**Table 1:** Demographics and covariates of the older adults in the CF and control groups.

Characteristics	Case group (*n* = 35)	Control group (*n* = 35)	t/χ2	*P*
Cognitive function (MoCA, scores)	12.74 ± 4.76	25.80 ± 2.07	14.88	<0.01
Physical frailty (EFS, scores)	8.63 ± 2.88	1.94 ± 1.11	12.81	<0.01
Age (years)	84.26 ± 7.99	66.80 ± 4.92	−11.005	<0.001
Males/females (numbers)	4/31	10/25	3.214	0.073
BMI (kg/m^2^)	22.71 ± 3.70	22.95 ± 4.22	0.248	0.81
Nutritional status (MNA-SF scores)	24.40 ± 3.54	27.19 ± 1.57	4.253	<**0.001**
Marital status (n, percentage)			8.741	0.003
Married	20 (57.14%)	31 (88.57%)		
Single	15 (42.86%)	4 (11.43%)		
Incomes (yuan/month)			0.325	0.850
**<**2,000	10 (28.57%)	8(22.86%)		
2,000 ~ 6,000	19 (54.29%)	20(57.14%)		
>6,000	6 (17.14%)	7(20.0%)		
Education (n, percentage)			28.812	<0.001
≤6 years	25(71.4%)	3(8.6%)		
>6 years	10(28.6%)	32(82.9%)		
Physical activity level (MET-min/week)	1398.66 ± 870.39	2423.30 ± 1520.00	3.461	0.01
Depression (n, percentage)			0.215	0.643
Yes	2(5.71%)	3(8.57%)		
No	33(94.29%)	32(91.43%)		
Current Smoking (n, percentage)			0.729	0.393
Yes	2(5.7%)	4(11%)		
No	33(94.3%)	31(88.6%)		
Current Drinking (n, percentage)			0.729	0.393
Yes	2(5.7%)	4(11%)		
No	33(94.3%)	31(88.6%)		
Medicines (n, percentage)			4.609	0.100
No	6 (17.1%)	12 (34.3%)		
1	16 (45.7%)	17 (48.6%)		
≥2	13 (37.1%)	6 (17.1%)		
Comorbidities (n, percentage)			2.143	0.143
Yes	24 (68.6%)	18 (51.4%)		
No	11 (31.4%)	17 (48.6%)		
Low sleep quality (PSQI, scores)			4.629	0.031
Yes (≥11 scores)	10(28.6%)	3(8.6%)		
No (<11 scores)	25(71.4%)	32(91.4%)		
Nutrient				
Protein (Kcal/d)	90.28 ± 35.55	104.50 ± 34.58	1.697	0.094
Fattiness (Kcal/d)	75.19 ± 29.96	90.70 ± 40.26	1.829	0.072
Carbohydrate (Kcal/d)	194.32 ± 50.21	216.36 ± 59.17	1.680	0.098
Dietary fiber (Kcal/d)	10.54 ± 3.57	14.26 ± 5.81	3.227	0.002

CF, cognitive frailty; BMI, Body mass index; MoCA, Montreal Cognitive Assessment; EFS, Edmonton Frailty Scale; IPAQ, International Physical Activity Questionnaire.

### 3.2 Receiver operating characteristic curves

**Figure 1:**
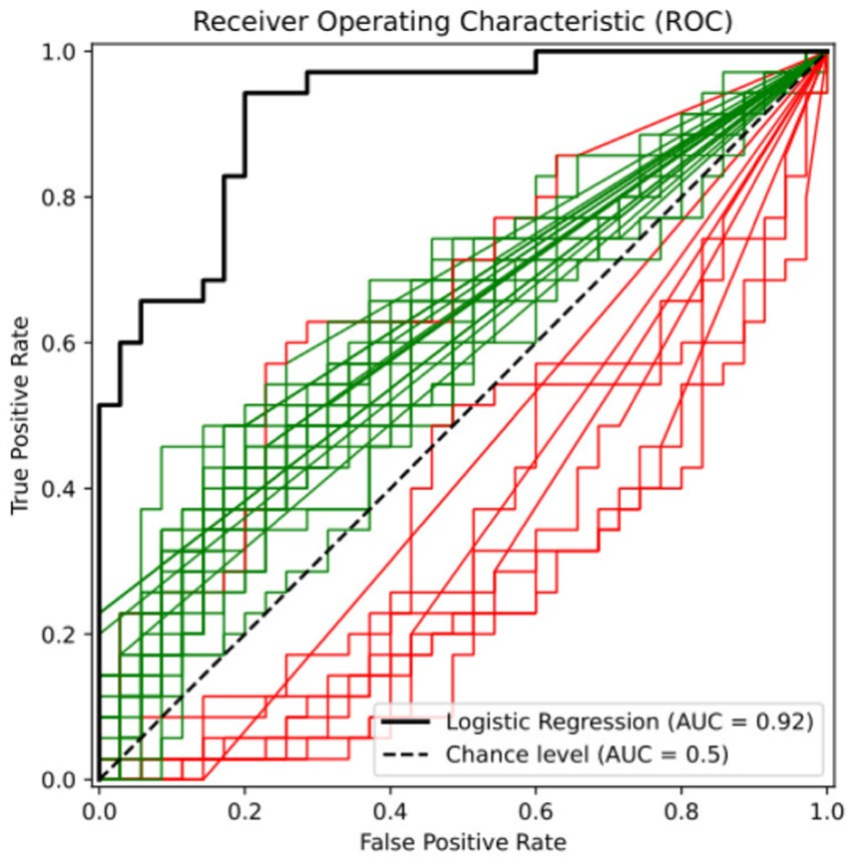
ROC curve of 35 bacterial genera for distinguishing CF. Black: logistic regression model; red: bacteria with higher abundance in the control group (KA) than in the case group (MA); green: bacteria with higher abundance in the MA group than in the KA group. In this figure shows that the ROC curves were fitted by the centered-log ratio transformed value of 35 bacterial genera of interest alone and together. The red curve represents bacteria with greater abundance in the KA group than in the MA group, and the green curve represents bacteria with greater abundance in the MA group than in the KA group. The black curve was fitted by logistic regression incorporating all 35 bacterial genera of interest as predictors. Overall, the 35 genera performed well in discriminating individuals with cognitive frailty from controls (area under the ROC curve (AUC) = 92.0%, sensitivity = 0.840, and specificity = 0.792), demonstrating the potential of the gut microbiota as a classification model.

Figure 1 shows that the ROC curves were fitted by the centered-log ratio transformed value of 35 selected bacterial genera based on the random forest algorithm alone and together. The red curve represents bacteria with greater abundance in the control group (KA) than in the case group (MA), and the green curve represents bacteria with greater abundance in the MA group than in the KA group. The black curve was fitted by logistic regression incorporating all 35 bacterial genera of interest as predictors. Overall, the 35 genera performed well in discriminating CF patients from controls [area under the ROC curve (AUC) = 92.0%, sensitivity = 0.840 and specificity = 0.792], demonstrating the potential of the gut microbiota as a classification model.

### 3.3 Biodiversity of the gut microbiota in the CF and control groups

**Figure 2:**
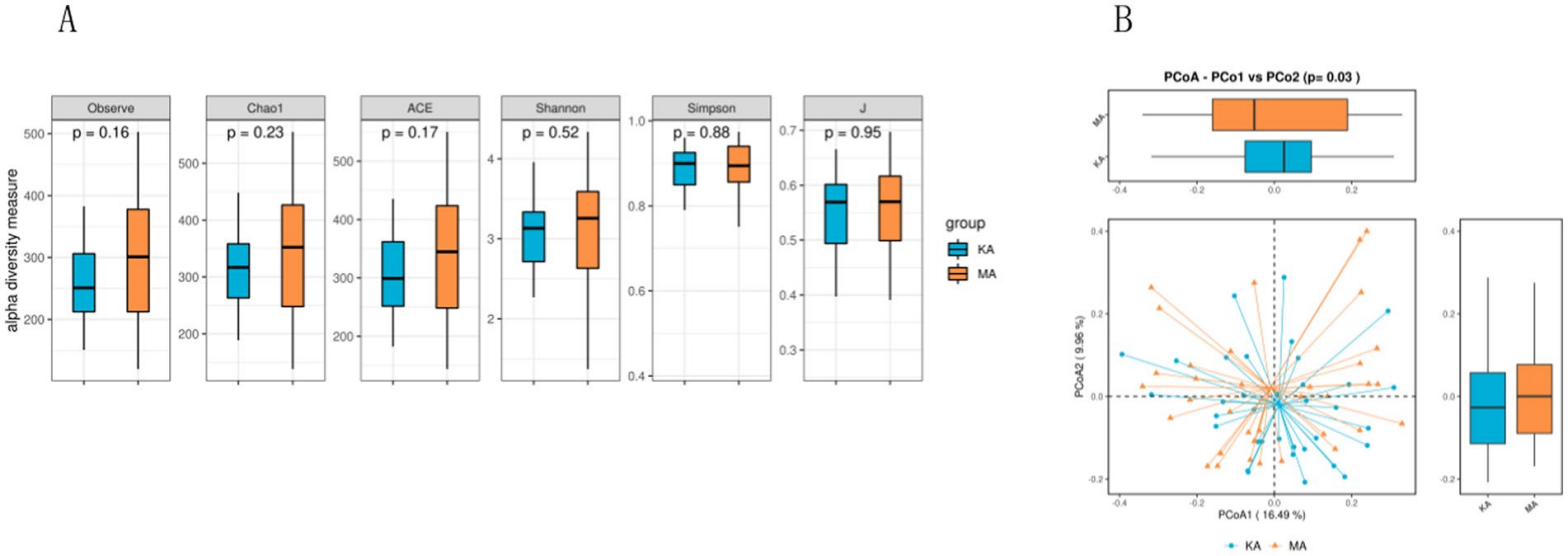
Comparison of *α* diversity and *β* diversity between the CF and control groups. MA represents the case (CF) group, and KA represents the control (non-CF) group. The alpha diversity measured via the observed, Chao 1, ACE, Shannon, Simpson, and J indices was similar between the two groups **(A)**. The horizontal coordinate indicates the sample groups, and the vertical coordinate indicates the alpha indices. The beta diversity presented by the PCoA was significantly different between the two groups **(B)**. Each point in the graph B is a sample, different colors indicate the groups of different experimental designs, and the closer the distance between the points is, the more similar the samples are. The PCoA was performed by using the distance matrix calculated from the species composition of the samples, and the horizontal and vertical axes represent the contributions of the first principal component (PC1) and the second principal component 2 (PC2), respectively. The two barplots in graph B represent the (PCoA-PCo1)/PCo2 values of the case or control group, respectively; and the *p* value is significance of comparison of the (PCoA-PCo1)/PCo2 values between two groups.

Figure 2 shows comparisons of biodiversity in the CF and control groups. There were no significant differences between the two groups in terms of alpha diversity, as measured by the observed, Chao 1, ACE, Shannon, Simpson, and J indices (Figure 2A) (*P* = 0.16, 0.23, 0.17, 0.52, 0.88, and 0.95, respectively), suggesting that the richness, diversity and uniformity of the gut microbiota were similar between the two groups. Beta diversity is used to assess the structure of the fecal microbiota and was characterized for samples using weighted UniFrac. The beta diversity presented by PCoA was significantly different between the two groups (*p* < 0.05) (Figure 2B), suggesting that there were significant differences in microbial community structure.

### 3.4 Discriminative taxa between the CF and control groups

**Figure 3:**
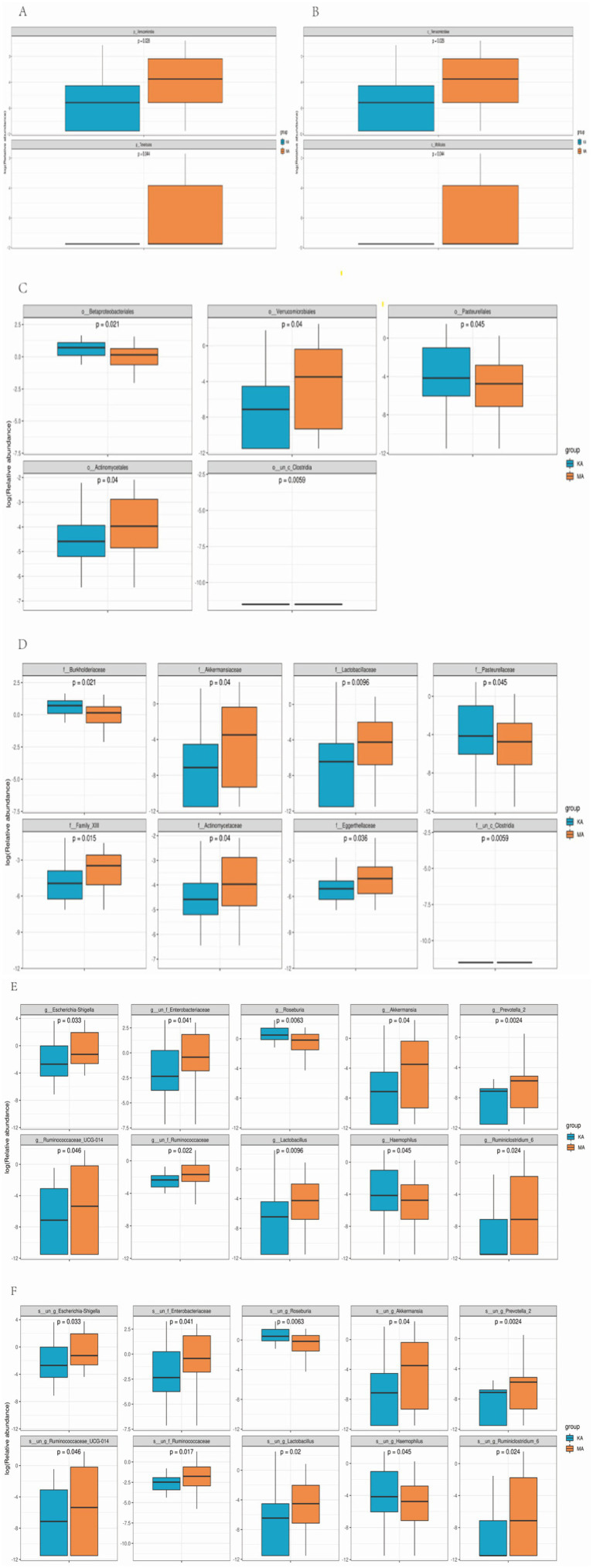
Comparison of the relative abundances at different gut microbiota levels between the CF and control groups. MA represents the CF group, and KA represents the control group. Box plot showing the significant differences at the phylum level **(A)**, class level **(B)**, order level **(C)**, family level **(D)**, genus level **(E)**, and species level **(F)** using the Kruskal–Wallis test and Wilcoxon signed rank test.

We further analyzed the specific changes in the gut microbiota between the CF and control groups. At the phylum level, *Verrucomicrobia* and *Tenericutes* were overrepresented in the CF group (Figure 3A) (both *p* < 0.05). The relative abundances of *Verrucomicrobiae* and *Mollicutes* at the class level were significantly higher in the CF group than in the control group (Figure 3B) (both *p* < 0.05). The relative abundances of *Verrucomicrobiales*, *Actinomycetales* and *Clostridia* were significantly higher, whereas the *Betaproteobacteriales* and *Pasteurellales* were significantly lower at the order level in the CF group than in the control group (Figure 3C) (both *p* < 0.05). At the family level, the relative abundances of *Akkermansiaceae*, *Lactobacillaceae*, *Family_XIII*, *Actinomycetaceae*, *Eggerthellaceae*, and *un_c_clostridia* were overrepresented; conversely, the relative abundances of *Burkholderiaceae* and *Pasteurellaceae* were lower in the CF group (Figure 3D) (both *p* < 0.05). At the genus level, the relative abundances of eight genera (e.g., *Escherichia-Shigella*, *Akkermansia*) were higher, whereas the relative abundances of *Roseburia* and *Haemophilus* were lower in the CF group than in the controls (Figure 3E) (all *p* < 0.05). At the species level, the relative abundance of eight species was higher, whereas two species were lower in the CF group than in the controls (Figure 3F) (both *p* < 0.05).

**Figure 4:**
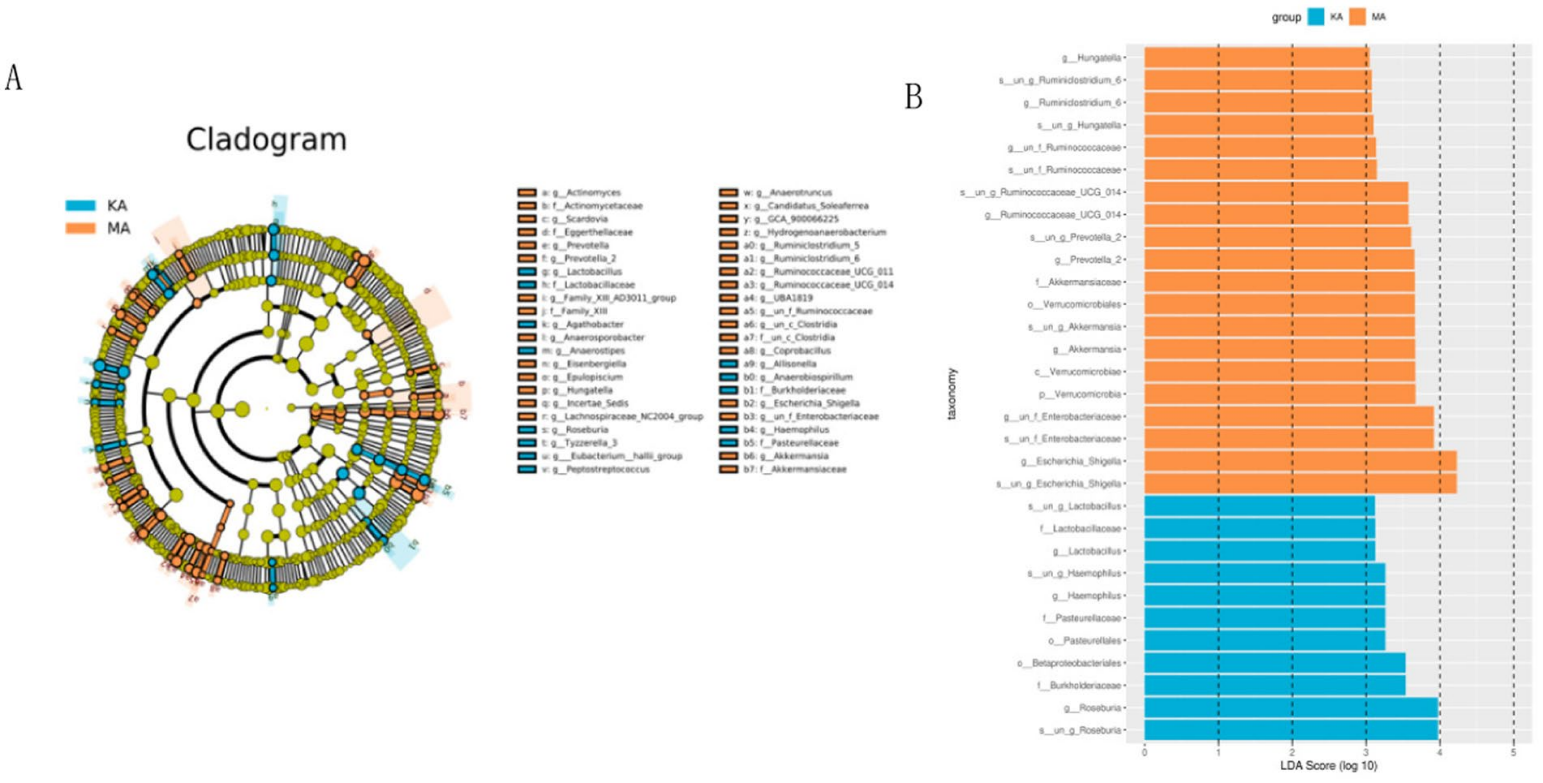
Discriminative taxa between the CF and control groups. MA represents the CF group, and KA represents the control group. Cladogram **(A)** and linear discriminant analysis of effect size (LEfSe) **(B)** showing biomarker taxa associated with CF. LEfSe analysis included individuals with cognitive frailty and healthy controls. The cladogram was generated using the Kruskal–Wallis test = 0.05, the Wilcoxon signed rank test = 0.05, and the LDA cutoff scores was set at the default value of 2.0.

LEfSe analysis was used to identify differences in the gut microbiota between the two groups. The cladogram revealed differences in the distribution of the gut microbiota in older adults between the CF and control groups (Figure 4A). The LDA bar plot revealed that the top four species in the control group were *s_un_g_Roseburia*, *Roseburia*, *Burkholderiaceae* and *Btaproteobacteriales*; the top four species in the CF group were *s_un_g_Escherichia-shigella*, *Escherichia-shigella*, *s_un_f_Enterobacteriaceae* and *g_un_f_Enterobacteriaceae* (Figure 4B).

### 3.5 Correlations between the gut microbiota and cognitive function or physical function

**Figure 5:**
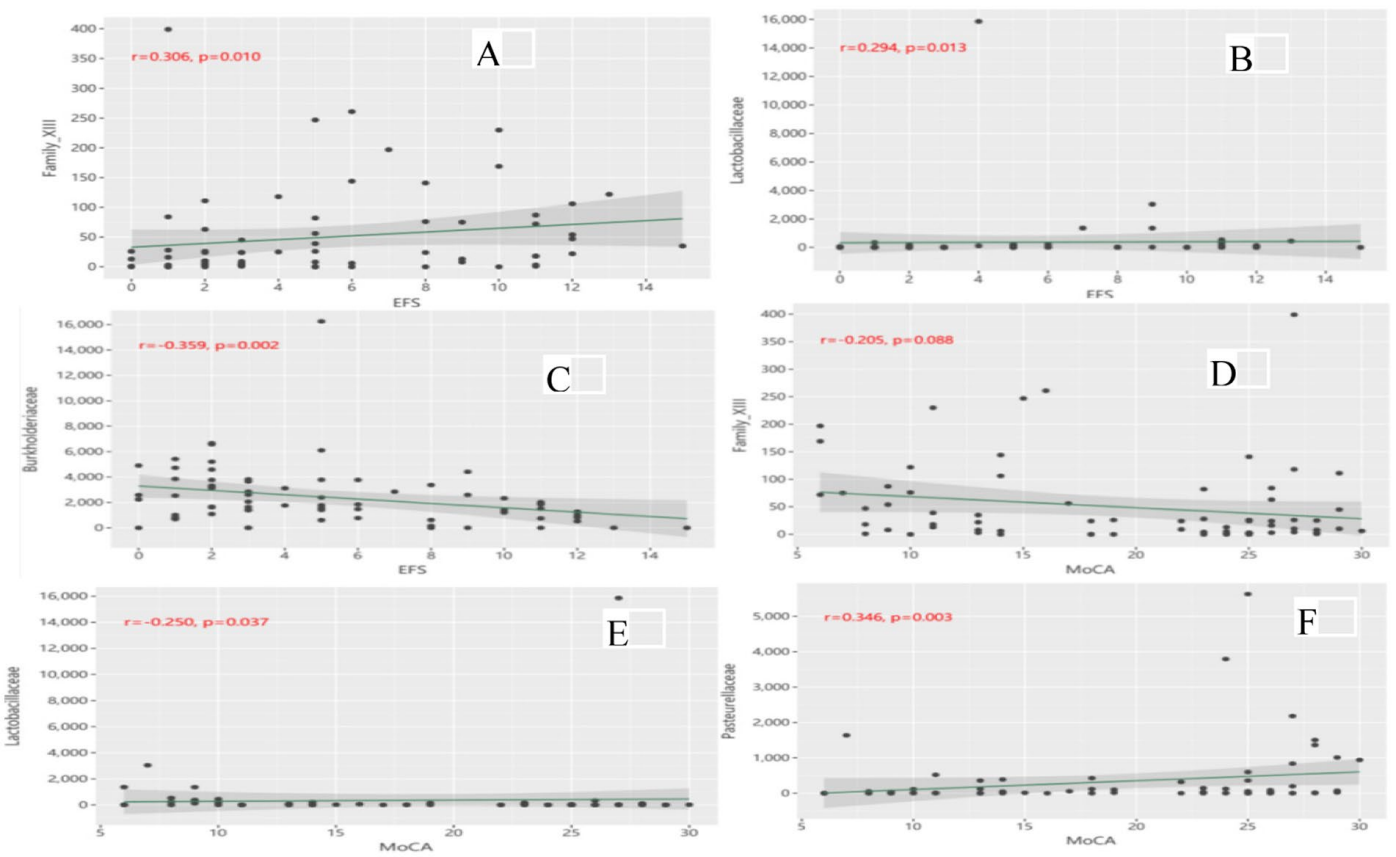
**(A)** Correlation of Family_XIII with EFS scores; **(B)** Correlation of Lactobacillaceae with EFS scores; **(C)** Correlation of Burkholderiaceae with EFS scores; **(D)** Correlation of Family_XIII with MoCA scores; **(E)** Correlation of Lactobacillaceae with MoCA scores; **(F)** Correlation of Pasteurellaceae with MoCA scores.

Figure 5 shows the partial correlations between gut microbiota abundance and cognitive and physical function at the family level, with adjustment for all imbalance variables between the two groups. At the family level, partial correlation analysis revealed that *Family_XIII* (Figure 5A) (*r* = 0.306, *p* = 0.010) and *Lactobacillaceae* (Figure 5B) (*r* = 0.294, *p* = 0.013) were positively correlated with EFS scores, whereas *Burkholderiaceae* (Figure 5C) (*r* = −0.359, *p* = 0.002) was negatively correlated with EFS scores. *Family_*XIII was not correlated with the MoCA score (Figure 5D) (*r* = −0.205, *p* = 0.088). *Lactobacillaceae* (Figure 5E) (*r* = −0.250, *p* = 0.037) was negatively correlated with the MoCA score, whereas *Pasteurellaceae* (Figure 5F) (*r* = 0.346, *p* = 0.003) was positively correlated with the MoCA score. No significant correlation was found at other microbiota levels.

**Figure 6:**
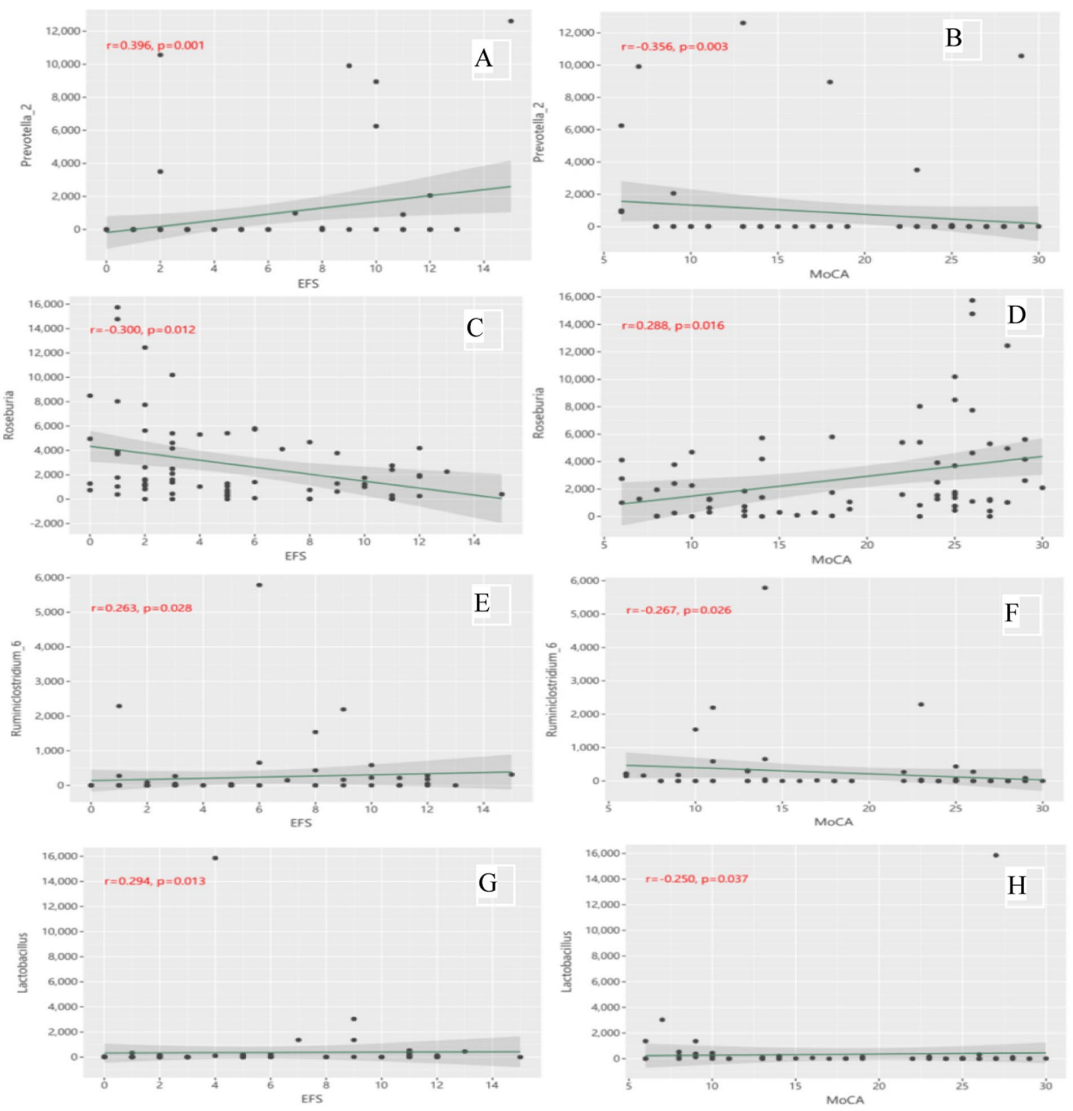
**(A)** Correlation of Prevotella_2 with EFS scores; **(B)** Correlation of Prevotella_2 with MoCA scores; **(C)** Correlation of Roseburia with EFS scores; **(D)** Correlation of Roseburia with MoCA scores; **(E)** Correlation of Ruminiclostridium_6 with EFS scores; **(F)** Correlation of Ruminiclostridium_6 with MoCA scores; **(G)** Correlation of Lactobacillus with EFS scores; **(H)** Correlation of Lactobacillus with MoCA scores.

Figure 6 shows the partial correlations between the gut microbiota abundance and cognitive and physical functions at the genus level, with adjustment for all imbalance variables between the two groups. At the genus level, *Prevotella_2* (Figure 6A) (*r* = 0.396, *p* = 0.001), *Ruminiclostridium_6* (Figure 6E) (*r* = 0.263, *p* = 0.028) and *Lactobacillus* (Figure 6G) (*r* = 0.294, *p* = 0.013) were positively associated with EFS scores, whereas *Roseburia* (Figure 6C) (*r* = −0.300, *p* = 0.012) was negatively associated with EFS scores. *Prevotella_2* (Figure 6B) (*r* = −0.356, *p* = 0.003), *Ruminiclostridium_6* (Figure 6F) (*r* = −0.267, *p* = 0.026) and *Lactobacillus* (Figure 6H) (*r* = −0.250, *p* = 0.037) were negatively correlated with MoCA scores, whereas *Roseburia* (Figure 6D) (*r* = 0.288, *p* = 0.016) was positively correlated with MoCA scores. No significant correlation was found at other microbiota levels.

## 4 Discussion

In this case–control study involving community-dwelling older adults, we observed a significant difference in *β* diversity of the gut microbiota between older adults with CF and those without CF, along with an altered microbial composition in the CF older adults. Specifically, two phyla, two classes, five orders, six families, and eight genera were more abundant in CF than in non-CF older adults, whereas two orders, two families, and two genera were less abundant. Among the microbiota enriched in the CF older adults, certain microbiota, sch as *Lactobacillaceae* were associated with poorer physical and cognitive function, but *Family_*XIII was linked only to poorer physical function. On the other hand, the CF-unabundant microbiota, such as *Burkholderiaceae*, correlated with better physical function, and *Pasteurellaceae* were associated with better cognitive function.

### 4.1 Comparison with previous studies

Currently, there is a lack of research that has directly compared the gut microbiota biodiversity of community-dwelling older adults with CF and without CF. However, several previous studies have reported significant differences in *α* diversity and/or β diversity of gut microbiota biodiversity between similar older adults, such as older adults with mild cognitive impairment compared to those with normal cognition, or frail older adults compared to non-frail older adults (23–26). This study identified a significant difference in the β-diversity of gut microbiota between older adults with CF and without CF, which is consistent with previous studies. The present study also revealed differences in the microbial composition at multiple taxonomic levels between older adults with CF and without CF. *Verrucomicrobia* and *Tenericutes* phyla, as well as *Verrucomicrobia* and *Mollicutes* classes, were more abundant in older adults with CF than in those without CF. Previous studies in frail or cognitively impaired older adults reported similar alterations in *Verrucomicrobia*, *Tenericutes*, and *Mollicutes*, supporting the link between gut dysbiosis and cognitive/physical decline. For instance, phylum and class of *Verrucomicrobia* bacteria have been reported to be more abundant in older adults with neurological impairment than in neurologically healthy older adults, and were significantly correlated with cognitive performance (27). Similarly, *Tenericutes* phylum has shown a negatively correlation with cognitive function in patients with schizophrenia (28). Both *Tenericutes* and *Mollicutes* bacteria have been associated with elevated levels of C-reactive protein, an acute inflammatory protein (29, 30).

This study also identified seven orders, eight families and 10 genera of gut microbiota that effectively differentiated older adults with CF from non-CF. Several CF-abundant gut microbiota, such as the families *Un_c_clostridia* and *Family_XIII* and the genera *Prevotella_2*, *Lactobacillus*, *Ruminiclostridium_6*, *un_f_Enterobacteriaceae* and *Ruminococcaceae_UCG-014*, were associated with poorer physical function, whereas the genera *Lactobacillaceae*, *Prevotella_2*, *Lactobacillus*, *Ruminiclostridium_6*, and *un_f_Enterobacteriaceae* were associated with poorer cognitive function. Conversely, the gut microbiota more abundant in the control group, such as *Roseburia*, *Haemophilus* and *Pasteurellaceae* genera, were associated with better cognitive function, while the families *Burkholderiaceae* and *Roseburia* were correlated with better physical function.

Moreover, many of these differentially abundant gut microbes have been previously associated with the pathophysiology of physical frailty or cognitive impairment. *Prevotella-2*, an inflammatory biomarker, is positively correlated with C-reactive protein or other inflammatory cytokines (31, 32). *Ruminococcaceae UCG-014* is linked with energy metabolism and inflammation (33). *Lactobacillus* has been found in higher abundant in patients with schizophrenia and chronic kidney disease (34). *Ruminiclostridium_6* abundance is positively correlated with inflammatory factors such as LPS, IL-6, TNF-*α*, and IL-17A (35). Additionally, this study observed a decrease in *Roseburia and Haemophilus* among older adults with CF. Both genera were positively correlated with better cognitive and physical function. Other previous research also supported the findings in our study, showing that the genus *Roseburia* was negatively correlated with the chronic inflammatory factor IL-6 (36), and *Haemophilus* abundance was positively correlated with cognition (37).

### 4.2 Potential mechanisms

Gut microbiota and their metabolites are increasingly recognized for their significant role in human health and disease. The complex interaction of microbiota-metabolites-host may directly or indirectly influence host metabolism and cognition. This occurs by integrating endocrine, neural, and immune signals via the microbiota-gut-brain (MGB) axis. The MGB axis facilitates communication via metabolites such as short chain fatty acids (SCFAs), and neurotransmitters such as dopamine, *γ*-amino butyric acid (GABA) and serotonin. These then modulate nutrient absorption and immune responses (38, 39). This axis also serves a dual function in defending against pathogens and modulating the host immune or nervous system, which has implications for age-related diseases such as frailty and neurodegenerative diseases. For example, SCFAs, metabolites produced by specific bacterial taxa in the human gut microbiota, are among the most abundant microbial byproducts, with butyrate being particularly notable. They play a critical role in alleviating inflammation, maintaining skeletal mass (40, 41), and exerting immunomodulatory and anti-inflammatory effects (42). They also regulate key physiological pathways within the nervous system (43). A growing body of research has demonstrated that SCFAs may help to mitigate neuroinflammation by influencing the structure and function of microglial cells, promoting the expression of neurotrophic factors, and upregulating tryptophan 5-hydroxylase 1. This has a beneficial effect on the integrity of the blood–brain barrier, resulting in the modulation of cognitive function (44, 45). In our study, we observed a higher abundance of *Roseburia* microbiota in non-CF older adults than in the CF older adults. Previous studies have identified *Roseburia* microbiota as a primary producer of SCFAs in the human colon (46), and an animal experiment showed that *R. hominis* could alleviate neuroinflammation by producing SCFAs such as propionate and butyrate (47).

### 4.3 Strengths and limitations

This study possesses several notable strengths. First, participants were recruited from among community-dwelling older adults with similar living environments and dietary habits. This enhances the generalizability of our findings to older adults in the real community living settings compared to those from clinics. Second, this study controlled for key factors known to influence the gut microbiota, such as medications, comorbidities and nutrients, and ensured balanced distributions of these variables between the CF and non-CF groups. Most importantly, this study identified 35 microbial genera that effectively differentiated the CF individuals from the non-CF older adults. This microbial signature demonstrated high accuracy in discriminating CF older adults from controls, with an area under the ROC curve of 92%, highlighting the potential of the gut microbiome to serve as a novel, non-invasive biomarker for predicting cognitive frailty in community-dwelling older adults.

Several limitations of this study should be acknowledged. First, although strict inclusion and exclusion criteria were applied to minimize confounding factors, the potential influence of unmeasured covariates cannot be ruled out. Second, due to the inherent limitations of the 16S rDNA sequencing method, we were unable to conduct a precise analysis of gut microbiota at the species level between the two groups. In addition, this study did not assess relevant metabolites or inflammatory factors. This limited our ability to investigate the underlying mechanisms by which the gut microbiota influences the pathological progression of CF. Finally, the cross-sectional design of this study precludes any causal inferences. Future longitudinal studies with larger sample sizes and multi-center designs are needed to elucidate the robust causal relationship of the gut microbiota with CF in community-dwelling older adults.

## 5 Conclusion

In conclusion, the results of the present study showed significant differences in the *β* diversity and distribution of the gut microbiota at the part phylum, class, order, family and genus levels between community-dwelling older adults with CF and those without CF, and the altered gut microbiota can differentiate CF from non-CF older adults. These findings support the role of the gut microbiota in the pathogenesis of CF in community-dwelling older adults. Further longitudinal studies with larger sample sizes are needed to elucidate their causal relationships.

## Data Availability

The datasets presented in this study can be found in online repositories. The names of the repository/repositories and accession number(s) can be found in the article/supplementary material.
